# The interplay between senescence, inflammation, and the immune system

**DOI:** 10.1101/gad.353125.125

**Published:** 2025-08-01

**Authors:** Jesús Gil

**Affiliations:** MRC Laboratory of Medical Sciences (LMS), London W12 0NN, United Kingdom; Institute of Clinical Sciences (ICS), Faculty of Medicine, Imperial College London, London W12 0NN, United Kingdom

**Keywords:** aging, senescence, inflammation

## Abstract

The past 40 years have witnessed significant progress in aging research. Although aging was once considered a stochastic process, it is now understood to be regulated by pathways and processes that can be dissected with modern cellular and molecular biology approaches. The aberrant accumulation of cells undergoing cellular senescence and an increase in chronic, sterile inflammation are two of those aging hallmarks. Here we discuss how these processes are connected and how the relationship between senescent cells and the immune system dictates the extent of inflammatory processes contributing to age-related dysfunction and disease.

## Abstract (precis)

In this Outlook, Gil discusses the mechanisms that contribute to the cross-talk between senescent cells and the immune system, including senescence-associated secretory phenotype (SASP) and nucleic acid sensing. Understanding the connection between cell senescence and chronic inflammation is vital to developing innovative therapeutic strategies to selectively eliminate senescent cells to mitigate aging and age-related disease.

Recent advances in the aging field were discussed during a 5 day meeting entitled “Senescence and Aging” at the Cold Spring Harbor Laboratory's 89th Symposium on Quantitative Biology. Many talks gravitated around the implications of cellular senescence for aging and aging-related disease. Cellular senescence is a stress response that limits the replicative potential of cells that are old, damaged, or stressed. Besides undergoing stable cell cycle arrest, senescent cells undergo numerous other phenotypic changes, including the expression of a bioactive secretome termed the senescence-associated secretory phenotype (SASP). In Dorcas Cummings’ lecture, “Zombie Cells at the Intersection of Cancer and Aging,” Scott Lowe introduced the senescent (“zombie”) cells to a broad audience. Just a year after two of the pioneers of the field (Leonard Hayflick [Serrano 2024] and Judy Campisi [Wiley et al. 2024]) passed away in 2024, Scott's talk beautifully explained in lay terms the nature and heterogeneity of these senescent “zombie” cells, how they contribute to disease, and the possibility of targeting them therapeutically.

Although senescence has essential roles in development, maintaining tissue homeostasis, or protecting against cancer initiation, the aberrant accumulation of senescent cells contributes to aging and fuels disease progression. This detrimental role of senescent cells is observed in a wide range of diseases, including fibrotic diseases, neurodegenerative diseases, and, paradoxically, cancer. For example, Fred Gage (Herdy et al. 2022) presented evidence showing how Alzheimer's disease (AD) brains are enriched in senescent neurons. By analyzing the expression of neurons of AD brains and taking advantage of protocols to produce aged induced neurons (iNs) from AD patients, Herdy et al. (2022) showed that these neurons with senescence features display a highly inflammatory profile. These senescent neurons are intrinsically dysfunctional, showing impaired electrical activity. More importantly, they can also condition other cells in the brain: Factors secreted by these neurons as part of the SASP can trigger reactive astrogliosis, which contributes to AD (Herdy et al. 2022). This key role of factors secreted by senescent neurons in mediating AD can be extended to other pathologies. The SASP is essential in directing the immune-mediated elimination of senescent cells but is also key in explaining how senescent cells contribute to aging and disease. Consequently, senescent cells and inflammation (and how the SASP is regulated) are intimately connected to the mechanisms explaining aging and disease.

Nucleic acid-sensing mechanisms, such as the cyclic GMP–AMP synthase/stimulator of interferon genes (cGAS/STING) pathway, are essential to activate innate immunity in response to infection and have been co-opted by senescent cells to induce the SASP. In response to infections, cGAS detects pathogenic cytosolic dsDNA to produce cyclic GMP–AMP (cGAMP) and activate STING. A talk by Andrea Ablasser reviewed the many mechanisms controlling cGAS/STING activation and regulation. In senescent cells, cGAS gets activated by self-DNA present in the cytosol. This cytoplasmic dsDNA can originate from aberrant blebbing in senescent nuclei, which produce cytoplasmic chromatin foci (CCFs) or DNA leaking from the mitochondria or are the by-product of mobilized transposons, a phenomenon that happens during senescence and aging.

Hanna Sladitschek-Martens et al. (2022) presented how the activation of cGAS/STING is also linked to other processes observed during aging, such as tissue decline. This can be controlled by the Yes-associated protein (YAP) and transcriptional coactivator with PDZ-binding motif (TAZ), which are key effectors regulating mechanosignaling. YAP/TAZ activity declines with age in stromal cells. YAP/TAZ activity maintains nuclear envelope integrity in part by controlling the expression of laminB1. A result of this age-related decline in YAP/TAZ activity is increased blebbing and production of CCFs that activate cGAS/STING (Sladitschek-Martens et al. 2022).

Another well-documented source of cytoplasmic dsDNA that accumulates during senescence and aging to activate cGAS/STING and IFN responses are retrotransposable elements such as those belonging to the L1 family (also referred to as LINE-1). A talk by John Sedivy (Martinez et al. 2024) showed how cGAS knockout mice exhibit an accelerated aging phenotype and increased levels of inflammation. This was surprising, because cGAS/STING activation itself induces inflammation. Interestingly, the increased inflammation observed in cGAS knockout mice was caused in part by chromatin changes that result in decreased DNA methylation on L1 transposons. This increased mobilization of L1 transposons is detected by other nucleic acid sensors (such as AIM2) and results in enhanced inflammation (Martinez et al. 2024).

This involvement of AIM2 in sensing dsDNA points to a broader role of other nucleic acid sensors in detecting damage-associated nucleic acids in pathological processes and aging. Jan Karlseder (Nassour et al. 2023) explained how replicative crisis, a process that eliminates cells with unstable telomeres and other chromatin aberrations, relies on a specialized program in which the Z-DNA binding protein 1 (ZBP1) senses telomeric repeat-containing RNA (TERRA) transcripts that are the by-product of dysfunctional telomeres. TERRA-bound ZBP1 forms filaments that activate another innate immune sensor, the mitochondrial antiviral signaling (MAVS) protein (Nassour et al. 2023). MAVS normally detects cytoplasmic dsRNA produced during viral infections. Manuel Serrano (López-Polo et al. 2024) also implicated MAVS as a damage sensor activated during senescence. Although mitochondrial DNA (mt-dsDNA) has been reported to activate cGAS/STING during senescence and to contribute to explaining SASP activation, mitochondrial double-stranded RNA (mt-dsRNA) is also produced in senescent cells and constitutes a key driver of the SASP, sensed by MAVS, that could be targeted therapeutically.

The above-described research helps to improve our understanding of the molecular mechanisms linking senescence, inflammation, and disease-associated tissue dysfunction during aging. However, it also suggests ways to intervene and target aging and its associated diseases. James Kirkland summarized the current state of clinical trials targeting senescent cells. Although these trials are still in their infancy, many interventions against cellular senescence or other hallmarks of aging are being pursued and supported through specific initiatives such as the Translational Geroscience Network of the National Institute on Aging.

Besides small molecule senolytics (that selectively kill senescent cells) and senomorphics (that, rather than killing senescent cells, reduce their associated inflammation), therapies that take advantage of the immune system to eliminate senescent cells are also actively being explored. Indeed, immune surveillance of senescent cells occurs naturally to eliminate the persistence of senescent cells in tissues. Therefore, immune system dysfunction (such as that observed in aging) or active immune evasion mechanisms can explain the aberrant accumulation of senescent cells observed in aging and disease.

Immune cell populations, including CD4^+^ and CD8^+^ T cells, macrophages, NK cells, or iNKT cells, have all been shown to be involved in eliminating different senescent cells. I presented work (Reen et al. 2025) in which we carried out functional screens to enhance the natural elimination of senescent cells by NK cells. Those studies unveiled how SMARCA4 inhibitors can increase the recruitment of NK cells by senescent cells. As a result, a PROTAC that targets SMARCA4 can be combined with cisplatin to reduce tumor size in an immunocompetent model of ovarian cancer (Reen et al. 2025). An alternative to achieve an immune-mediated elimination of senescent cells was presented by Scott Lowe and Corina Amor (Amor et al. 2020). They have taken advantage of the fact that senescent cells express high levels of the urokinase-type plasminogen activator receptor (uPAR) on their cell surface. By developing chimeric antigen receptor (CAR) T cells targeting uPAR, they devised a new way to eliminate senescent cells. This selective elimination of senescent cells extends the survival of mouse models of lung cancer and improves tissue homeostasis in models of liver fibrosis (Amor et al. 2020). Moreover, Corina Amor (Amor et al. 2024) showed that such CAR-T cells can be administered in a prophylactic fashion; a single administration has long-lasting effects, and anti-uPAR CAR-T cells can improve physical fitness and ameliorate metabolic dysfunction in aged mice or mice kept on a high-fat diet, further speaking of the promise of such an approach.

When the meeting was coming to an end, I reflected that my first visit to Cold Spring Harbor was more than 20 years ago, for the 2002 Meeting on “Molecular Genetics of Aging.” The passing of time could be noticed in how the participants of that early meeting, who were also present at this Symposium (including me), have aged. However, perhaps the most notorious change is how what in 2002 were nascent links between the aging process and genetic pathways have solidified and how novel strategies have emerged to target aging and its associated diseases. At the core of those, the connection between senescent cells, inflammation, and the immune system has not only provided important mechanistic insights about the aging process but has also served to unveil therapeutic targets that should become tangible in years to come.
